# Antimicrobial resistance of *Vibrio cholerae* from sub-Saharan Africa: A systematic review

**DOI:** 10.4102/ajlm.v7i2.778

**Published:** 2018-12-06

**Authors:** Yahaya Mohammed, Aaron O. Aboderin, Iruka N. Okeke, Adebola T. Olayinka

**Affiliations:** 1 Department of Medical Microbiology and Parasitology, Faculty of Basic Clinical Sciences, College of Health Sciences, Usmanu Danfodiyo University, Sokoto, Nigeria; 2 Department of Medical Microbiology and Parasitology, College of Health Sciences, Obafemi Awolowo University, Ile-Ife, Nigeria; 3 Department of Pharmaceutical Microbiology, Faculty of Pharmacy, University of Ibadan, Ibadan, Nigeria; 4 Department of Medical Microbiology and Parasitology, Faculty of Medicine, Ahmadu Bello University, Zaria, Nigeria

## Abstract

**Background:**

The World Health Assembly adopted the Global Action Plan on Antimicrobial Resistance, which includes improving the knowledge base through surveillance and research. Noteworthily, the World Health Organization has advocated a Global Antimicrobial Resistance Surveillance System to address the plan’s surveillance objective, with most African countries enrolling in or after 2017.

**Aim:**

The aim of this article was to review prior data on antimicrobial resistance of *Vibrio cholerae* from sub-Saharan Africa with a view for future control and intervention strategies.

**Methods:**

We used the Preferred Reporting Items for Systematic Review and Meta-Analysis (or ‘PRISMA’) guidelines to search the PubMed and African Journals Online databases, as well as additional articles provided by the Nigeria Centre for Disease Control, for articles reporting on the antibiotic susceptibility of *V. cholerae* between January 2000 and December 2017.

**Results:**

We identified 340 publications, of which only 25 (reporting from 16 countries within the sub-Saharan African region) were eligible. The majority (20; 80.0%) of the cholera toxigenic *V. cholerae* isolates were of the serogroup O1 of the El Tor biotype with Ogawa and Inaba serotypes predominating. Resistance was predominantly documented to trimethoprim-sulphamethoxazole (50% of the studies), ampicillin (43.3% of the studies), chloramphenicol (43.3% of the studies) and streptomycin (30% of the studies). Resistance mechanisms were reported in 40% of the studies.

**Conclusion:**

Our results demonstrate a documented antimicrobial resistance of *V. cholerae* to multiple antibiotic classes, including cell wall active agents and antimetabolites with evidence of phenotypic/genotypic resistance to fluoroquinolones.

## Introduction

*Vibrio cholerae* are Gram-negative and curved bacilli. Certain members of this species are associated with a severe acute watery diarrhoea that is the most distinctive sign of a clinical condition called cholera.^1^ There exist numerous *V. cholerae* serogroups, but the O1 and O139 strains stand out as the major agents for the major outbreaks of cholera globally. The serogroup O139 is majorly restricted to some parts of Asia; however, serogroup O1 *V. cholerae*, further subdivided into the El Tor and classical biotypes, are distributed worldwide.^2^

The *V. cholerae* O1 El Tor biotype was responsible for the seventh cholera pandemic, which started in Indonesia and spread rapidly to Bangladesh, India, Iran and Iraq.^3^ Cholera was imported to Africa in the 1970s from these countries during this seventh pandemic. It entered from West Africa from where it spread to East, Central and South Africa.^4^

*V. cholerae* virulence and drug resistance evolved during the course of the seventh pandemic and a new variant cholera biotype emerged.^2^ This variant is called the ‘hybrid’ or ‘atypical’ biotype and it has mixed markers of the classical and El Tor biotypes. Hybrid *V. cholerae* have the El Tor biotype, but with the non-El Tor *ctxB* toxigenic allele. This atypical El Tor biotype is associated with higher virulence and more widespread antibiotic resistance.^2^

Atypical *V. cholerae* carry mobile genetic elements like the integrative/conjugative elements (ICEs), which are capable of self-transfer and integration into host chromosomes, facilitating rapid spread and stable acquisition.^5^

Recently, the occurrence of new variant pathogenic strains of *V. cholerae* has been attributed to new CTX prophage rearrangements.^6^ Resistant *V. cholerae* have disseminated globally and now threaten the effective treatment and control of cholera, especially in the low and middle-income countries.^1,5^ Recent evidence suggests that cholera is exacting a very high burden on the African continent in this era.^6^ However, few data are available about the nature and extent of outbreaks or the properties of strains.^7^ Multi-country or global studies generally have less input from Africa.^8^

Upon commissioning from the Nigeria Centre for Disease Control (NCDC), we set out to review data on the antimicrobial resistance of *V. cholerae* from publications done in sub-Saharan Africa, in order to provide evidence that may serve as a yardstick for future control programmes and interventions.

## Methodology

### Overview of study protocol

This systematic review was done using the Preferred Reporting Items for Systematic Reviews and Meta-Analysis (or ‘PRISMA’) guidelines.^9^ The protocol for the study was developed in conjunction with the NCDC panel of experts and a more detailed version of the protocol is available on request.

### Search strategy

We searched Medline using PubMed for articles published in English between 01 January 2000 and 31 December 2017 with the search terms ‘antimicrobial resistance’, ‘antibiotic resistance’, ‘*Vibrio cholerae*’ and the names of the individual countries of sub-Saharan Africa. Additional searches were done in the African Journals Online database, using an additional search term of ‘antimicrobial susceptibility’. We also scanned a list of articles obtained from the NCDC to select eligible articles that conformed with our search terms.

### Study selection criteria

Articles were included for this review provided they reported on the antimicrobial susceptibility profile of *V. cholerae* isolates from clinical specimens in sub-Saharan Africa and were published between January 2000 and December 2017. We included articles irrespective of whether the isolates were obtained as part of an outbreak investigation or from a hospital-based site using cross-sectional survey, provided they were from human specimens.

### Selection procedure

**FIGURE 1:**
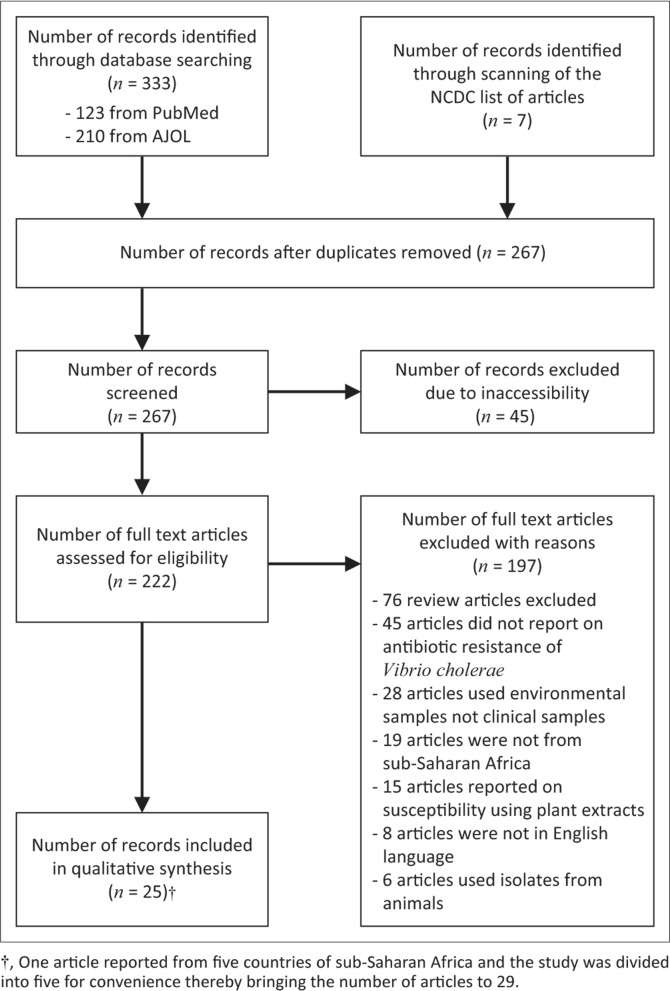
Summary of study selection procedure.

The titles and abstracts of all search results were listed and were thereafter reviewed to identify papers for full text review. The selection procedure is outlined in Figure 1. Forty-five papers were excluded, because all attempts to secure full text versions were unsuccessful. Names of authors from articles were not blinded before or after the full text review. We used predetermined inclusion and exclusion criteria to select papers for full review. Papers selected for full review after abstract review were retrieved as full manuscript papers through PubMed, HINARI, from the NCDC or by personal communication with the corresponding authors. Seventy-six review articles were excluded, and 45 papers did not report on the susceptibility pattern of *V. cholerae* and were also excluded. Twenty-eight papers were on environmental samples; hence, they were excluded. Nineteen articles were not from sub-Saharan Africa and 15 articles evaluated susceptibility to plant extracts and, consequently, they were all excluded. Eight articles were in French and six articles used isolates from animal sources and they were all excluded.

### Data extraction

A database was created in which the study name, study period, susceptibility pattern, biochemical properties, genes and virulence factors of the *V. cholerae* isolates from countries of sub-Saharan Africa were recorded where applicable (Table 1). We could not adequately carry out a quantitative study, because most of the susceptibility patterns of the isolates were not recorded in actual numerical values. Most of the studies also did not report on the quality control procedure they used and some of the studies relied only on molecular detection of resistance genes.

**TABLE 1:** Characterisation of eligible articles that sought resistance of *Vibrio cholerae* from countries in sub-Saharan Africa.

Study number	Study name	Year	Ref	Study location	Proportion of resistance to cell wall active agents (%)	Proportion of resistance to fluoroquinolones (%)	Proportion of resistance to nucleic acid inhibitors (%)	Proportion of resistance to protein synthesis inhibitors; 30S (%)	Proportion of resistance to protein synthesis; 50S (%)
1	Ceccarelli D	2011	6	Angola	100	100	100	100	100
2	Kaas RS	2010	10	Chad	NA	100	100	100	100
3	Kacou-N’douba A	2012	11	Cote d’Ivoire	25	0	100	0	100
4	Smith AM†	2015	12	Cote d’Ivoire	0	50	100	0	100
5	Miwanda B	2015	13	DRC	NA	0	100	NA	100
6	Smith AM†	2015	12	DRC	0	50	100	25	0
7	Opintan JA	2008	15	Ghana	100	50	100	100	100
8	Eibach D	2016	14	Ghana	100	25	100	0	0
9	Dalsgaard A	2000	4	Guinea Bissau	100	NA	100	100	100
10	Smith AM†	2015	12	Guinea Bissau	100	0	100	0	100
11	Urassa WK	2000	16	Kenya	100	100	0	100	100
12	Scrascia M	2006	17	Kenya	NA	0	100	0	100
13	Sang WK	2012	18	Kenya	100	50	NA	0	100
14	Dromigny J-A	2002	19	Madagascar	100	100	NA	100	NA
15	Smith AM†	2015	12	Mozambique	100	50	100	100	100
16	Dengo-Baloi LC	2007	20	Mozambique	100	50	100	50	50
17	Smith AM	2007	21	Namibia	0	0	100	0	0
18	Okeke IN	2001	22	Nigeria	NA	NA	100	0	100
19	Opajobi SO	2004	23	Nigeria	100	0	NA	0	50
20	Quilici M-L	2010	24	Nigeria	50	100	100	0	25
21	Marin MA 2013	2013	25	Nigeria	NA	100	100	NA	25
22	Marin MA 2014	2014	26	Nigeria	NA	0	100	0	25
23	Sambe-Ba B	2017	30	Senegal	NA	NA	100	0	NA
24	Ismail H	2008	31	South Africa	100	50	100	100	50
25	Moyo SJ	2011	32	Tanzania	100	0	0	100	100
26	Mercy N	2014	33	Tanzania	NA	0	100	0	0
27	Smith AM†	2015	12	Togo	0	50	100	0	0
28	Mwansa JCL	2006	34	Zambia	NA	NA	NA	100	NA
29	Chiyangi H	2017	35	Zambia	0	75	100	100	0

† , This article reported from five countries of sub-Saharan Africa (Reference 12).

Ref, reference number from this study; DRC, Democratic Republic of the Congo; NA, not available.

### Attempt to reduce bias

An attempt to reduce bias within studies and between individual studies was done. The review was also conducted in a group with materials, articles and the papers double-checked by at least two members of the group.

### Analysis approach

The extracted data were reported as outlined by the authors using the susceptibility categorisation of the pathogens into sensitive, intermediate and resistant. We reported on the biochemical characteristics of the *V. cholerae* in terms of serogroup, biotype and serotype. We also reported on the clinical diagnosis for isolates in studies where diagnosis was stated. We also highlighted the use of molecular methods, genotyping or virulence features for the characterisation of isolates wherever such data were available.

## Results

### General characteristics of the studies included in the analysis

Our search generated 267 articles after removal of duplicates. During abstract review, we excluded 242 articles, because they did not meet our inclusion criteria. Twenty-five articles were included in the final analysis.

One article reported resistance of *V. cholerae* from five sub-Saharan African countries and was therefore divided into five for convenience. In all, the articles obtained reported on 16 of the 47 countries within the sub-Saharan African region. One study (3.4% for each) was obtained from each of the following countries: Angola, Chad, Madagascar, Namibia, Senegal, South Africa and Togo. Two studies (6.9% for each) were from each of Cote d’Ivoire, Democratic Republic of the Congo, Ghana, Guinea Bissau, Mozambique, Tanzania and Zambia. Three (10.3%) studies were from Kenya and five (17.2%) were from Nigeria.

Twenty-four (82.8%) of the studies reported serogroup O1 as the only serogroup, while two (6.9%) studies reported the O1 serogroup coexisting with the non-O1/non-O139 serogroup, but even in those studies the O1 serogroup dominated. Three (10.3%) studies did not report on the serogroup status. None of the studies reported the O139 serogroup from any country in the region of sub-Saharan Africa.

Twenty two (75.9%) studies reported the El Tor biotype, while one (3.4%) study reported the existence of the El Tor and the atypical El Tor biotype. Six (20.7%) of the studies did not biotype their isolates. There was no report of the classical biotype from any of the studies.

Eight (27.6%) studies reported the Ogawa serotype as the predominant serotype, three (10.3%) studies reported on Inaba existing alone and six (20.7%) reported the Ogawa/Inaba coexisting together. The coexistence of Inaba/Ogawa/Hikojima was reported from one (3.4%) study. Eleven (37.9%) of the studies did not report on any of the serotypes.

Seventeen studies detected or confirmed cholera toxin and toxin-co-regulated pilus genes (*ctxB, ctxA* and *tcpA*).

Table 1 shows the characteristics of eligible articles that investigated resistance of *V. cholerae* from sub-Saharan Africa. Resistance was documented to trimethoprim-sulphamethoxazole (50% of the studies), ampicillin (43.3%), chloramphenicol (43.3%), streptomycin (30%), nalidixic acid (30%), nitrofurantoin (26.7%), ceftriaxone (20%), spectinomycin (10%), sulfonamide (6.7%), penicillin G (6.7%) and cloxacillin (3.3%). The antibiotics to which susceptible strains were reported were: tetracycline (46.7% of the studies), amoxicilin/clavulanic acid (6.7%), florfenicol (3.3%), azithromycin (3.3%), imipenem (3.3%), ciprofloxacin (3.3%), ofloxacin (3.3%) and erythromycin (3.3%).

Mutations in antibiotic resistance determinants (*gyrA, parC, floR, strA, and strB)* were detected in nine (31.1%) of the studies, while twenty (68.9%) studies did not conduct genotypic studies on the isolates.

The *ICEVchAng2* and *ICEVchInd5* were reported from seven (24.1%) of the studies while the other twenty-two (75.9%) studies did not perform this genotypic analysis.

### Summary of the resistance studies on the *Vibrio cholerae* isolates from the various studies

The average prevalence of resistance to cell wall active agents by the *V. cholerae* organisms from 20 studies was 68.8% (100–0%). However, 25 studies reported on the resistance to fluoroquinolones with their total average of 44.0% (100–0%), while the average prevalence of resistance to inhibitors of nucleic acid, predominantly the sulphonamide and co-trimozaxole, was 92.0% from 25 studies (Table 1).

Twenty-seven studies reported 43.5% (100–0%) prevalence of resistance to protein synthesis inhibitors of 30S subunit, while the average prevalence of resistance to protein synthesis inhibitors of 50S subunit (Table 1) was 62.5% (100–0%) from 26 eligible studies.

### Specific characteristics of the studies included in the analysis

In Angola, Ceccarelli et al. performed a retrospective study on *V. cholerae* O1 El Tor strains responsible for the 2006 outbreak. The isolates were resistant to all the major groups of antimicrobials tested and they demonstrated the appearance of a novel *V. cholerae* epidemic variant in Africa with a new CTXΦ arrangement previously described only in the Indian subcontinent.^6^ Kaas et al. investigated the 2010/2011 *V. cholerae* outbreak in Lake Chad basin around Cameroon. The outbreak strains were resistant to all the antimicrobials tested and, in addition, possessed the integrative conjugative element *ICEVchInd5*. This is said to be clonal and clustered, distant from the other African strains.^10^ Kacou-N’douba et al. documented resistance to chlorampenicol and cotrimoxazole during a cholera epidemic in 2011 from Cote d’Ivoire.^11^

Smith and colleagues characterised *V. cholerae* O1 from Cote d’Ivoire, Democratic Republic of the Congo, Guinea Bissau, Mozambique and Namibia. All the isolates were of Ogawa serotype and positive for the *ctxA* gene. There was generalised resistance to nalidixic acid, chloramphenicol and cotrimoxazole in all isolates from the five countries.^12^ In Democratic Republic of the Congo, Miwanda et al. documented resistance to cotrimoxazole, erythromycin and chloramphenicol. However, no resistance to fluoroquinolones was reported from this study.^13^ In two separate studies from Ghana, Eibach et al.^14^ and Opintan et al.^15^ documented resistance to trimethoprim-sulphamethoxazole, ampicillin and nalidixic acid. Dalsgaard and colleagues^4^ from Guinea Bissau demonstrated resistance to ampicillin, aminoglycosides, cotrimoxazole and tetracycline. Only colistin remained effective from their study. They also demonstrated that resistant isolates possessed a multi-resistance transmissible plasmid that encoded trimethoprim (*dhfrXII*) and aminoglycoside resistance (*ant(3”)-1a*).^4^

In Kenya, Urassa et al.^16^ documented resistance to ciprofloxacin, tetracycline, ampicillin, erythromycin and chloramphenicol by *V. cholerae* during two separate outbreaks.^16^ Another study from Kenya by Scrascia et al.^17^ showed resistance of *V. cholerae* to chloramphenicol, streptomycin and cotrimoxazole.^17^

Sang and colleagues^18^ in Kenya were able to demonstrate resistance to ciprofloxacin, tetracycline, ampicillin, erythromycin and chloramphenicol among *V. cholerae* isolates.^18^ Dromigny and colleagues^19^ conducted a study in Madagascar in 2002 among *V. cholerae* isolates and documented resistance to tetracycline, ampicillin, nalidixic acid and nitrofurantoin.^19^ Dengo-Baloi et al.^20^ performed a study in Mozambique to determine the antibiotic resistance patterns of *V. cholerae* O1 Ogawa. The isolates were resistant to ampicillin, azithromycin, sulphamethoxazole, nalidixic acid and nitrofurantoin. Genes for cholera toxin (*ctxA, rstR2, tcpA*) and the virulence factors of *ICEVchBan9* and *ICEVchInd5* were elaborated.^20^

Smith from Namibia^21^ characterised isolates of *V. cholerae* from a 2006/2007 outbreak and the isolates were all resistant to trimethoprim, sulphamethoxazole and streptomycin. The isolates possessing either the *SfiI* or *NotI* digestion sites were further analysed using advanced molecular techniques and it was demonstrated that they all have the same origin.^21^ Okeke and colleagues^22^ investigated an outbreak of acute gastroenteritis from Niger state, north-central Nigeria, where eight *V. cholerae* organisms were isolated. They all had the O1-serogroup and El Tor biotype. All of them were sensitive to tetracycline but resistant to trimethoprim, sulphonamide, spectinomycin and chloramphenicol.^22^

Opajobi et al.^23^ detected 34 strains of *V. cholerae* in Jos University Teaching Hospital over a one-year period. They were all of the O1 serogroup, El Tor biotype and Inaba serotype. They were all resistant to chloramphenicol, ampicillin, cloxacillin and penicillin G, but sensitive to tetracycline, ofloxacin and erythromycin.^23^

A study done by Quilici et al.^24^ using the *V. cholerae* isolates from the September/October 2009 outbreak of acute watery diarrhoea in north-eastern Nigeria and northern Cameroon implicated the serogroup O1 of the El Tor biotype and Ogawa serotype as the causative serotypes. The toxigenic genes of *ctxA* and *ctxB* were elaborated, in addition to detected mutations in the genes responsible for quinolone resistance. The *ctxB* gene was similar to the one detected in India. All of them were resistant to trimethoprim-sulphamethoxazole, ciprofloxacin, sulphonamide and nalidixic acid. All the isolates were resistant to tetracycline, but moderately susceptible to chloramphenicol and ampicillin.^24^ In 2013, Marin and colleagues^25^ described *V. cholerae* that were isolated from cases of acute watery diarrhoea outbreaks in Nigeria from 2009 to 2010. They reported that these toxigenic *V. cholerae* isolates were mostly of O1 serotype, and that atypical El Tor strains with the integrative conjugative element (ICE) of the sulfamethoxazole and trimethoprim (SXT) element, *gyrA*, cholera toxin (CTX) phage and cytidine triphosphate (CTP) synthetase clusters showed reduced susceptibility to ciprofloxacin and chloramphenicol.

Another study by Marin et al.^26^ in 2014 characterised the whole genome of 13 strains of *V. cholerae* that were obtained from the 2010 outbreak. They all harboured an ICE (*ICEVchNig1*) that was characterised and shown to possess genes for trimethoprim, sulfamethoxazole, streptomycin and chloramphenicol resistance. They were found to have the same gene content and gene order with similar elements detected over 20 years ago in Haiti,^27^ Angola^6^ and Bangladesh.^28^ Dutilh and colleagues^29^ used an innovative model to characterise the genomic variation of microbial genome with specific reference to *V. cholerae* isolates obtained worldwide with Nigeria inclusive. They were able to outline mobile functions of phages, prophages, transposable elements, and plasmids. They constructed a phylogenetic tree that revealed that the *V. cholerae* strain isolated in 2010 from Nigeria was closely related to strains already circulating in Nepal and Haiti.^29^

In Senegal, the study by Sambe-Ba et al.^30^ identified atypical El Tor *V. cholerae* O1 Ogawa that were resistant to streptomycin and cotrimoxazole. Ismail and colleagues^31^ from South Africa characterised a multi-resistant *V. cholerae* with resistance to ampicillin, cotrimoxazole, nalidixic acid, tetracycline, kanamycin and streptomycin. The isolates were positive for the SXT element, had quinolone resistance-determining mutations in the genes encoding GyrA (*Ser83-Ile*) and ParC (*Ser85-Leu*) and produced *TEM*-63-β-lactamase.^31^

A study in Tanzania by Moyo et al.^32^ documented resistance to ampicillin, amoxicillin-clavulanate, erythromycin, chloramphenicol, tetracycline, gentamicin and cephalothin. Another study in Tanzania by Mercy et al.^33^ identified resistance of *V. cholerae* to furazoline, trimethoprim-sulphamethoxazole, polymyxin-B and streptomycin. The cholera virulence determinant genes *ctxA, tcpA, ctxB* and *rtxC* were also elaborated. In Zambia, Mwansa and colleagues^34^ documented resistance of *V. cholerae* to trimethoprim, sulphamethoxazole, tetracycline and furazolidine. The cholera toxin and the virulence genes *ctxA, rstR2, rfbO1* and *tcpA* were also elaborated.^34^ Chiyangi et al.,^35^ also from Zambia, detected resistance to cotrimoxazole, nalidixic acid and nitrofurantoin.^35^

## Discussion

Cholera outbreaks have been ongoing within sub-Saharan African countries for the past four decades. Unfortunately, the specific strains responsible and their antibiotic resistance patterns are not well studied and elucidated.^36^ This consequently impacts negatively on the control programmes for cholera across the continent.

Despite our extensive database searches, we could only find a few articles that exclusively met our inclusion criteria of reporting antibiotic resistance profiles of *V. cholerae* from sub-Saharan Africa. This reflects a worrisome neglect of research on *V. cholerae* resistance trends from sub-Saharan Africa, despite the high prevalence of *V. cholerae* and its almost seasonal occurrence.^29,37^

We were able to retrieve some of the identified full text reviews from major databases by personally contacting the corresponding authors, which we recommend for researchers from developing countries like ours. There is a changing pattern of *Vibrio cholerae* serogroups and biotypes responsible for cholera outbreaks worldwide.^2^ During the seventh pandemic, the typical El Tor strain was responsible for outbreaks in Asia, Caribbean countries and Africa. However, studies have now highlighted the fact that the multi-drug resistant atypical El Tor and non-O1/non-O139 *V. cholerae* strains are the major drivers worldwide with sub-Saharan Africa included.^2^ The study done by Marin et al.^22^ signifies that the 2009/2010 outbreaks were caused by a highly multi-drug resistant atypical El Tor strain carrying major virulence determinants.^22^

Our review revealed only *V. cholerae* isolates of the O1 and the non-O1/non-O139 serogroup with absence of the O139 serogroup from sub-Saharan Africa. This is consistent with literature evidence that reflects the occurrence of the *V. cholerae* O139 to be mostly restricted to Bangladesh and parts of India.^37^

None of the studies in our review detected the ‘classical’ biotype of *V. cholerae*. The classical biotype has ceased to be implicated in cholera outbreaks globally, has been replaced by the El Tor since after the sixth pandemic and is now mostly restricted to Bangladesh.^38^

The stand-alone existence of the Hikojima serotype was not detected by any of the studies from our review. This is not surprising as the Hikojima serotype is rare and contains all the major antigens (A, B and C). One current hypothesis is that the Hikojima serotype is an unstable serotype that represents a transitional state between Ogawa to Inaba serotype.^39^

*V. cholerae* displayed an increasingly complex resistance phenotype to various antimicrobial drugs from our review. The majority of the studies we reviewed did not state the guidelines they followed in conducting the antimicrobial susceptibility testing; nevertheless, they reported variable levels of resistance to the fluoroquinolones. The existence of quinolone resistance-determining mutations in *gyr*A and *par*C in most of the isolates studied provides a genetic basis for fluoroquinolone resistance. The isolated *Vibrio cholerae* strains that harboured the fluoroquinolone resistant genes were resistant to multiple antimicrobial agents, which has important implications for the antimicrobial-based epidemic control strategies that are still the mainstay within countries in sub-Saharan Africa.^40^

An increasing trend of resistance to cotrimoxazole was observed from many studies. Kacou-N’douba^11^ and Smith et al.^12^ documented resistance to chloramphenicol and cotrimoxazole from their studies in Cote d’Ivoire, Democratic Republic of the Congo, Guinea Bissau, Mozambique and Namibia.^11,12^ This is worrisome, because, until now, cotrimoxazole was considered the drug of choice against *V. cholerae*.

This study provides support for the more recently advocated vaccine-based strategies, which have a better chance of reducing outbreak size and case fatality rates. As access to cholera vaccine stockpiles is dependent on laboratory confirmation of cholera, public health laboratory strengthening is essential.^41^

In Angola, Ceccarelli et al. demonstrated the appearance of a novel *V. cholerae* epidemic variant in Africa with a new CTXΦ arrangement previously described only on the Indian subcontinent.^6^ Similarly, Quilici et al.^24^ identified a strain of *V. cholerae* from Nigeria with resistant *ctxB* clones that are similar to a strain identified earlier in India. This possibly indicates a trans-continental transmission of resistant organisms and this has implication for global health for appropriate international control.^42^

The finding of transferable resistance to almost all of the antibiotics commonly used to treat cholera was documented from many studies. Some of this was documented by Ceccarelli,^1^ Kaas,^2^ Dalsgaard^4^ and Ismail.^31^ This is of great public health concern and a cause of alarm for the continent.

This finding also highlights the need to develop Africa’s capacity in terms of national reference laboratories, because the use of serotyping and bio-typing is inadequate for tracking the origin and clonality of *V. cholerae* isolates. Genotypic analysis, multi-locus sequence analysis, pulse field gel electrophoresis or whole genome sequence analyses are needed to track clonality. Unfortunately, these methods are more advanced and only available in a few reference laboratories within the continent.^4^

### Limitations

The most appropriate pictorial representation for meta-analytic data is the forest plot. However, we could not construct one because the studies we included did not provide the component data that is essential for a forest plot.

### Conclusion

Antimicrobial resistance exists among *V. cholerae* isolates from sub-Saharan Africa and includes the most feared fluoroquinolone resistance variety as well as resistance to the cell wall active agents and antimetabolites. The volume of research from countries in sub-Saharan Africa on antimicrobial resistance trends in *V. cholerae* needs to be expanded and better explored. Guidelines on antimicrobial chemotherapy and standardisation of antimicrobial susceptibility testing need to be strictly adhered to.
